# Sensitive and Selective Spectrophotometric Determination of Gabapentin in Capsules Using Two Nitrophenols as Chromogenic Agents

**DOI:** 10.1155/2011/619310

**Published:** 2011-06-22

**Authors:** Sameer A. M. Abdulrahman, Kanakapura Basavaiah

**Affiliations:** Department of Chemistry, University of Mysore, Manasagangotri, Mysore 570006, India

## Abstract

Two simple and selective spectrophotometric methods have been proposed for the determination of gabapentin (GBP) in pure form and in capsules. Both methods are based on the proton transfer from the Lewis acid such as 2,4,6-trinitrophenol (picric acid; PA) or 2,4-dinitrophenol (2,4-DNP) to the primary amino group of GBP which works as Lewis base and formation of yellow ion-pair complexes. The ion-pair complexes formed show absorption maximum at 415 and 420 nm for PA and 2,4-DNP, respectively. Under the optimized experimental conditions, Beer's law is obeyed over the concentration ranges of 1.25–15.0 and 2.0–18.0 **μ**g mL^−1^ GBP for PA and 2,4-DNP methods, respectively. The molar absorptivity, Sandell's sensitivity, detection and, quantification limits for both methods are also reported. The proposed methods were applied successfully to the determination of GBP in pure form and commercial capsules. Statistical comparison of the results was performed using Student's *t*-test and F-ratio at 95% confidence level, and there was no significant difference between the reference and proposed methods with regard to accuracy and precision. Further, the validity of the proposed methods was confirmed by recovery studies via standard addition technique.

## 1. Introduction

Gabapentin (GBP), chemically known as 1-(aminomethyl) cyclohexaneacetic acid [1], is freely soluble in water as well as in both basic and acidic aqueous solutions. GBP was originally developed for the treatment of epilepsy; it is currently also used against neuropathic pain. Although its exact mechanism of action is yet unknown, it is believed to involve voltage-gated N-type calcium ion channels in the central nervous system, reducing calcium influx into the nerve terminals [2]. In this way, the nerves become less excitable, reducing the release of other neurotransmitters. 

Several techniques are available for the determination of GBP in pharmaceutical preparations and include fluorimetry using sequential injection [2], spectrofluorimetry [3, 4], chemiluminometry [5], high performance liquid chromatography (HPLC) [6–11], capillary electrophoresis [12, 13], potentiometric sensor [14], voltammetry [15], visible spectrophotometry [16–20], UV spectrophotometry [20, 21], and automated spectrophotometry using piezoelectric pumping [22]. 

To the best of our knowledge, there are five reports on the use of visible spectrophotometry for the determination of GBP in pharmaceuticals. Abdellatef and khalil [16] have reported three methods based on three different reactions involving the use of vanillin in the presence of McIlvain buffer pH 7.5, ninhydrin reagent in DMF medium, and p-benzoquinone in ethanol medium. The method reported by Al-Zehouri et al. [17] was based on the condensation of GBP with acetylacetone and formaldehyde according to Hantzsch reaction. The charge transfer complexation reactions of GBP as n-electron donor with various acceptors such as iodine, chloranil, chloranilic acid, DDQ, TCNQ, and TCNE were reported by Salem [18]. The reaction of GBP with ninhydrine in DMF medium has served as the basis of spectrophotometric assay reported by Galande et al. [19]. Siddiqui et al. [20] have reported two different reactions involving ninhydrin in methanol medium and TCNQ in acetonitrile. However, many of the above methods suffered from one or other disadvantage like poor sensitivity, measurements done at shorter wavelengths, heating or cooling step, the use of expensive chemical and/or complicated experimental setup as can be seen from Tables 1 and 2. 

The reagents under study, that is, 2,4,6-trinitrophenol (picric acid; PA) and 2,4-dinitrophenol (2,4-DNP) have numerous applications as analytical reagents, and they have been used for the spectrophotometric determination of many drugs in pharmaceutical formulations [23–26]. 

This paper describes the application of PA and 2,4-DNP to the spectrophotometric determination of GBP in bulk drug as well as in capsules. The proposed methods are based on the formation of ion-pair complexes as a result of a proton transfer from PA or 2,4-DNP to the primary amino group of GBP. The proposed methods have been demonstrated to be superior to many reported methods with respect to speed, simplicity, sensitivity and cost effectiveness, and can be adopted by the pharmaceutical laboratories for industrial quality control.

## 2. Experimental

### 2.1. Instrument

A Systronics model 106 digital spectrophotometer (Systronics, Ahmedabad, Gujarat, India) equipped with 1 cm matched quartz cells was used for all absorbance measurements.

### 2.2. Materials

Pharmaceutical grade gabapentin (GBP) which is reported to be 99.5% pure was received from Sun Pharmaceuticals, Mumbai, India. The following pharmaceutical preparations were purchased from commercial sources in the local market and subjected to analysis: Gabantin-100 (100 mg GBP per capsule) from Sun Pharma Sikkim, Ranipool, East Sikkim, India and Gabapin-300 (300 mg GBP per capsule) from Intas Pharmaceuticals, Dehradun, India.

### 2.3. Reagents and Chemicals

All reagents used were of analytical reagent grade, and HPLC grade organic solvents were used throughout the investigation.

A 2.0 g L^−1^ PA and 2.0 g L^−1^ 2,4-DNP solutions were prepared separately in dichloromethane for use in method A and method B, respectively. A stock standard solution containing 100 *μ*g mL^−1^ GBP was prepared by dissolving 10 mg of pure drug in 2.0 mL methanol and diluting to the mark in a 100 mL calibrated flask with acetonitrile. The stock standard solution was diluted appropriately with acetonitrile to get working concentrations of 25 and 40 *μ*g mL^−1^ GBP for use in method A and method B, respectively.

### 2.4. Assay Procedure

#### 2.4.1. Method A (Using PA)

Different aliquots (0.25–3.0 mL) of a standard GBP (25.0 *μ*g mL^−1^) solution were accurately transferred into a series of 5 mL calibrated flasks using a microburette, and the total volume was adjusted to 3.0 mL by adding adequate quantity of acetonitrile. One milliliter of 2.0 g L^−1^ PA solution was added to each flask and the mixture was diluted to the volume with acetonitrile and mixed well. The absorbance of each solution was measured at 415 nm against a reagent blank after 10 min.

#### 2.4.2. Method B (Using 2,4-DNP)

Aliquots (0.25–2.25 mL) of a standard GBP (40 *μ*g mL^−1^) solution were accurately transferred into a series of 5 mL calibrated flasks, as described above. To each flask was then added 0.75 mL of 2.0 g L^−1^2,4-DNP, and the content was diluted to the volume with acetonitrile and was mixed well. After 10 min, the absorbance was measured at 420 nm against a reagent blank prepared simultaneously.

#### 2.4.3. Procedure for Capsules

The content of ten capsules each containing 100 or 300 mg of GBP was weighed. An accurately weighed quantity equivalent to 10 mg of GBP was transferred into a 100 mL calibrated flask and dissolved in 2.0 mL methanol followed by the addition of 60 mL acetonitrile. The solution was shaken thoroughly for about 15–20 min, diluted to the mark with acetonitrile, mixed well, and filtered using a Whatman No. 42 filter paper. The first 10 mL portion of the filtrate was discarded, and a suitable aliquot of the filtrate (100 *μ*g mL^−1^ GBP) was diluted to get the working concentrations of 25 and 40 *μ*g mL^−1^ GBP for analysis by methods A and B, respectively, as described above.

#### 2.4.4. Procedure for the Selectivity Study

Selectivity was evaluated by both placebo blank analysis and recovery studies. A placebo blank, the commonly employed excipients added to the formulations, consisting of 30 mg starch, 20 mg lactose, 20 mg acacia, 20 mg calcium gluconate, 50 mg talc, 30 mg magnesium stearate, and 20 mg sodium alginate was prepared as described under “Procedure for capsules” and then subjected to analysis. 

A synthetic mixture was prepared by adding 10 mg of pure GBP to 50 mg of the above mentioned placebo blank, and the mixture was homogenized. Following the same procedure for capsules, the synthetic mixture solution was prepared, and a suitable quantity was subjected for analysis by both the methods.

### 2.5. Stoichiometric Relationship

Job's method of continuous variations of equimolar solutions was employed to establish the stoichiometry of the formed ion-pair complexes. The solutions equivalent to 1.46 × 10^−4^ and 1.17 × 10^−3^ M GBP were prepared by dissolving the calculated amount of GBP in a minimum amount of methanol and diluting to volume with acetonitrile. Further, 1.46 × 10^−4^ M PA and 1.17 × 10^−3^ M 2,4-DNP solutions were prepared in dichloromethane. A series of solutions was prepared in which the total volume of GBP and reagent was kept at 2.5 mL in a total volume of 5 mL. The solutions were mixed well; the volume was completed to the mark with both acetonitrile and dichloromethane keeping the ratio of the two solvents as 1 : 1 in each flask. The absorbance of the resulting solutions was measured after 10 min at the respective wavelengths of maximum absorbance (*λ*_max_) against the blank consisted of (1 : 1) acetonitrile and dichloromethane.

## 3. Results and Discussion

### 3.1. Absorption Spectra

**Figure 1:**
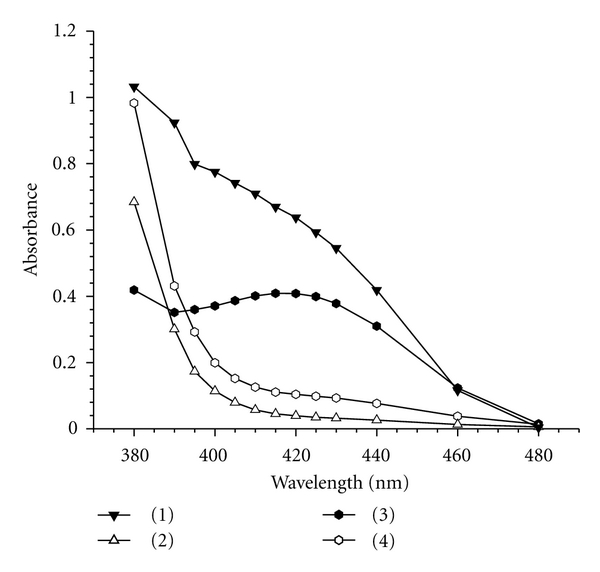
Absorption spectra of (1) sample versus reagent blank (7.5 *μ*g mL^−1^ GBP)-method A; (2) reagent blank-method A; (3) sample versus reagent blank (8.0 *μ*g mL^−1^ GBP)-method B; (4) reagent blank-method B.

The reaction of PA or 2,4-DNP as Lewis acids with GBP as Lewis base results in the formation of an intense yellow colored products. The absorption spectra of the yellow colored products were recorded at 380–480 nm against the corresponding blank solutions. The resulted yellow colored ion-pair complexes showed maximum absorbance at 415 and 420 nm for GBP-PA and GBP-2,4-DNP, respectively, (Figure 1). 

### 3.2. Reaction Mechanism

**Figure 2:**
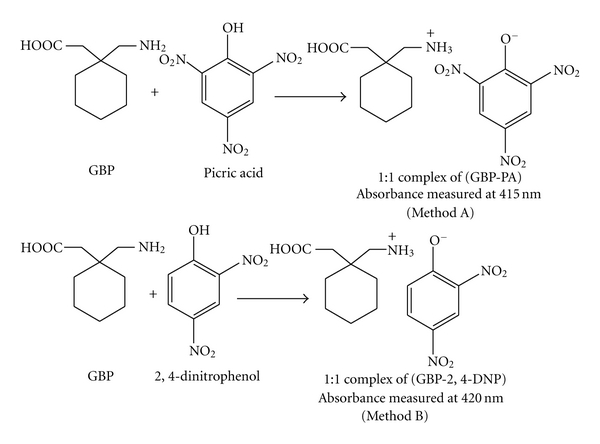
The probable reaction mechanism for the formation of GBP-PA and GBP-2,4-DNP ion-pair complexes.

The chemistry used in the proposed methods is based on the proton-transfer from the hydroxyl group of the Lewis acid such as PA or 2,4-DNP to the primary amino group of the Lewis base, GBP, resulting in the formation of yellow colored ion-pair complexes. Higuchi and Brochmann-Hanssen [27] have reported that the basic aliphatic amines form salts with picric acid in organic solvents which are much more intensely colored than picric acid itself, and this is due to the fact that the negatively charged picrate ion (phenolate ion) is intensely colored (yellow), where as the undissociated form, as it exists in neutral or acidic solvents is very lightly colored. Similarly, Saito and Matsunaga [28] reported that when the aliphatic amine is combined with a polynitrophenol, the fore field produces an acid-base interaction which leads to the formation of true phenolate by proton transfer. The possible reaction mechanisms are proposed and illustrated in Figure 2. 

### 3.3. The effect of Different Experimental Variables

Some variables which found to affect the intensity of the resulting ion-pair complexes were optimized to achieve maximum analytical sensitivity and adherence to Beer's law. 

#### 3.3.1. Effect of Reagent Concentration

**Figure 3:**
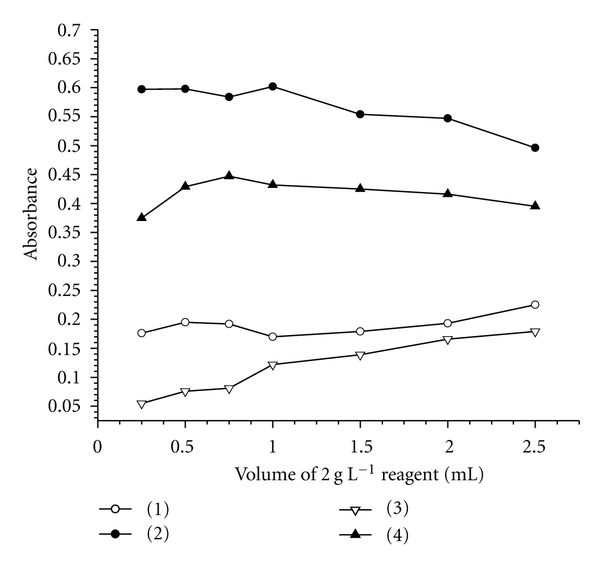
Effect of reagents concentrations on the color development: (1) corresponding blank-method A; (2) sample versus corresponding blank (8.0 *μ*g mL^−1^ GBP)-method A; (3) corresponding blank-method B; (4) sample versus reagent blank (10.0 *μ*g mL^−1^ GBP)-method B.

The effect of the reagent concentration on the intensity of the formed yellow colored complexes at the selected wavelengths was studied by measuring the absorbance of solutions containing fixed concentrations of 8.0 and 10.0 *μ*g mL^−1^ GBP and different amounts (0.25–2.5 mL) of the reagents PA and 2,4-DNP for methods A and B, respectively. The results showed that 1.0 mL of 2.0 g L^−1^ PA and 0.75 mL of 2.0 g L^−1^2,4-DNP solutions were optimum for the production of maximum and reproducible color intensity (Figure 3). 

#### 3.3.2. Effect of Solvent

The effect of different solvents to prepare GBP solution was restricted since its solubility is limited to a few organic solvents such as methanol and acetonitrile. Even though GBP is freely soluble in methanol and less soluble in acetonitrile, methanolic solution of GBP couldn't be used in the assay because methanol gave intense yellow color with PA and 2,4-DNP. So, to prepare the stock solution, GBP was first dissolved in a minimum amount of methanol and subsequently diluted with acetonitrile. The results showed that the effect of methanol used to prepare the stock solution of GBP was negligible. 

**Figure 4:**
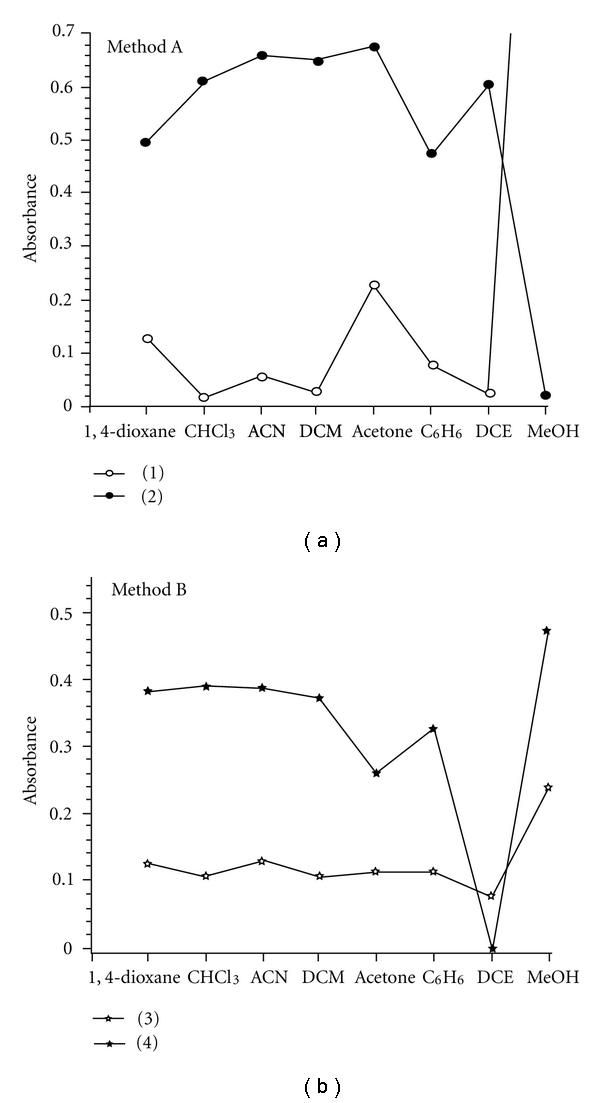
Effect of solvents on the color development: (1) corresponding blank-method A; (2) sample versus corresponding blank (7.5 *μ*g mL^−1^ GBP)-method A; (3) corresponding blank-method B; (4) sample versus reagent blank (7.5 *μ*g mL^−1^ GBP)-method B.

In order to select the suitable solvent to prepare the PA and 2,4-DNP solutions, both reagents were prepared separately in different solvents such as 1,4-dioxane, chloroform, acetonitrile, dichloromethane, acetone, benzene, dichloroethane, and methanol. Then, the reaction of GBP with PA or 2,4-DNP was carried out in the solvents mentioned above, and the absorbance of each solution was measured at the selected wavelengths against the corresponding blank. In method A, the results showed that the corresponding blank in methanol gave maximum absorbance against methanol compared with the sample for the same solvent against the corresponding blank, and dichloromethane was the ideal solvent which was finally used for the preparation of PA solution (Figure 4). In method B, as shown in Figure 4, both chloroform and dichloromethane were found suitable to be used for the preparation of 2,4-DNP solution, but the latter was preferred due to the high stability of the measured species compared with the same in chloroform medium. Also, the effect of the diluting solvent was tested for both methods, and the results showed that acetonitrile was the ideal diluting solvent to achieve maximum sensitivity in both the methods. 

#### 3.3.3. Effect of Reaction Time and Stability of the Measured Species

The optimum reaction time was determined by following the absorbance of the developed color upon the addition of PA or 2,4-DNP solution to the GBP solution at room temperature. For both methods, the reaction was found to be complete and quantitative when the reaction mixture was allowed to stand for 10 min, and any delay in the absorbance measurements of the yellow ion pair complexes had no effect on the reaction stoichiometry which was determined to be 1 : 1 (GBP: reagent) for the ranges studied. The ion-pair complexes of GBP with PA and 2,4-DNP, which were used for quantitation of the drug, were found to be stable up to 48 and 24 hrs, respectively.

### 3.4. Composition of the Ion-Pair Complex

**Figure 5:**
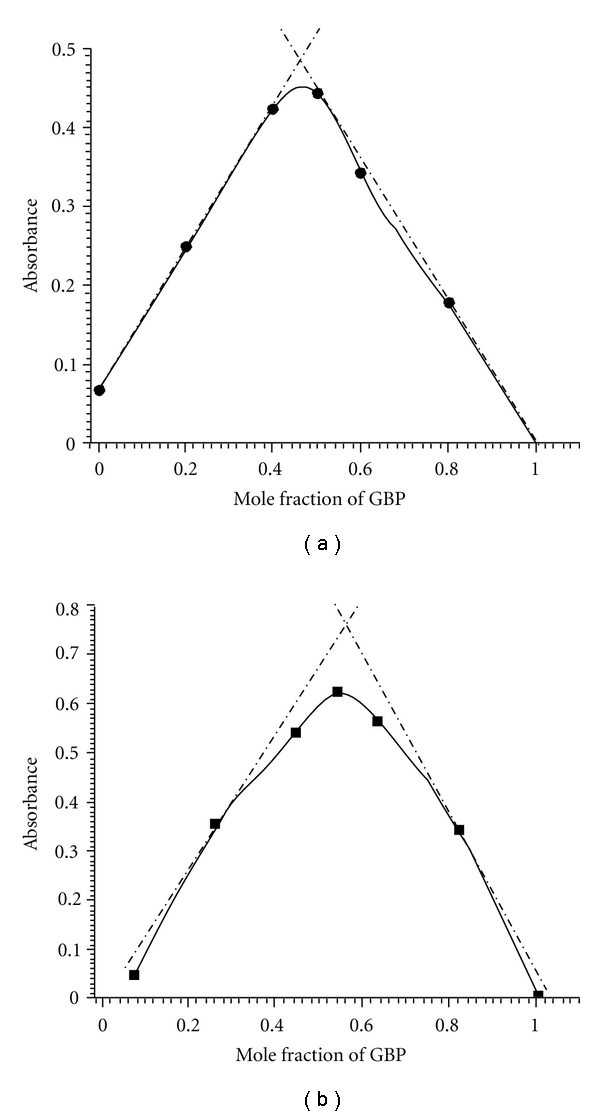
Job's Continuous-variations plots (a) GBP + PA; (b) GBP + 2,4-DNP.

Job's continuous variations graph for the reaction between GBP and PA or 2,4-DNP shows that the interaction occurs on an equimolar basis via the formation of ion-pair complexes (Figure 5). The plot reached a maximum value at a mole fraction of 0.5 which indicated that a 1 : 1 (GBP : PA) and (GBP : 2,4-DNP) ion-pair complexes are formed through the electrostatic attraction between positive protonated GBP and nitrophenolate anions. This finding was anticipated by the presence of one basic or electron-donating centre (−NH_2_) in the GBP. The conditional stability constants (K_f_) of the ion-pair complexes were calculated [29] from the data of continuous variations method and found to be 5.73 × 10^7^ and 4.54 × 10^5^ for GBP-PA and GBP-2,4-DNP complexes, respectively. The high values of K_f_ confirm the expected high stabilities of the formed ion-pair complexes. This parameter is strongly dependent on the nature of the used acceptor including the type and number of electron-withdrawing substituents to it such as nitro groups [30]. This is the reason for the high value of K_f_ of GBP-PA ion-pair complex compared with the same for 2,4-DNP. 

### 3.5. Method Validation

#### 3.5.1. Linearity

Under the optimized experimental conditions for GBP determination, the standard calibration curves for GBP with PA and 2,4-DNP were constructed by plotting absorbance versus concentration. The linear regression equations were obtained by the method of least squares, and the Beer's law range, molar absorptivity, Sandell's sensitivity, correlation coefficient, standard deviation of intercept (S_a_), standard deviation of slop (S_b_), limits of detection, and quantification for both methods are summarized in Table 3. 

The limit of detection (LOD) and limit of quantification (LOQ) for the proposed methods were calculated using the following equations [31]:

(1) $$\text{LOD}=\frac{3.3\times \sigma }{\text{S}},\;\;\text{LOQ}=\frac{10\times \sigma }{\text{S}},$$

where *σ* is the standard deviation of *n* replicate determinations (*n* = 8 for method A and *n* = 6 for method B) under the same conditions as for the sample analysis in the absence of the analyte, and S is the sensitivity, namely, the slope of the calibration graph.

#### 3.5.2. Accuracy and Precision

In order to evaluate the precision of the proposed methods, solutions containing three different concentrations of GBP were prepared and analyzed in seven replicates during the same day (intraday precision) and five consecutive days (interday precision), and the results were summarized in Table 4. The low values of the percentage relative standard deviation (RSD ≤ 2.34% for intraday) and (RSD ≤ 2.59% for interday) indicate the high precision of the proposed methods. Also, the accuracy of the proposed methods was evaluated as percentage relative error (RE%), and from the results shown in Table 4, it is clear that the accuracy is good (RE ≤ 3.60%). 

#### 3.5.3. Selectivity

The selectivity of the proposed methods for the analysis of GBP was evaluated by analysis of placebo blank solution as shown under “Procedure for capsules”, and the resulting absorbance readings in both methods were same as reagent blank, inferring no interference from the placebo. Noninterference from placebo was further confirmed by carrying out recovery study from synthetic mixture which with percent recoveries of 99.42 ± 2.05 and 98.56 ± 2.43 for method A and method B, respectively. These results confirm the selectivity of the proposed methods in the presence of the commonly employed excipients added to the formulations.

#### 3.5.4. Robustness and Ruggedness

The evaluation of the method robustness was done by making small incremental changes in two experimental variables, reagent volume and reaction time, and performing the analysis under the altered experimental conditions. The effect of the changes on the absorbance reading of the resulted complexes in both methods was studied and found to be negligible confirming the robustness of the proposed methods. Method ruggedness was expressed as  %R.S.D of the same procedure applied by three analysts and also by a single analyst performing analysis on three different instruments. The results presented in Table 5 showed that no statistical differences between different analysts and instruments suggesting that the proposed methods were rugged. 

#### 3.5.5. Analysis of Pharmaceutical Formulations

The proposed methods were applied to the determination of GBP in two representative capsules Gabantin-100 and Gabapin-300. The results obtained are compiled in Table 6 and were compared with those obtained by the reference method [21] by means of Student's *t*-test for accuracy and *F*-tests for precision at 95% confidence level. The reference method consisted of the measurement of the absorbance of the aqueous extract of the capsules at 210 nm. As can be seen from Table 6, the calculated *t* and *F* values at 95% confidence level did not exceed the tabulated values of 2.78 and 6.39, respectively, indicating that there were no significant differences between the proposed methods and the reference method with respect to accuracy and precision.

### 3.6. Recovery Study

The accuracy and validity of the proposed methods were further confirmed by the standard addition procedure. Preanalyzed capsule powder (Gabantin-100 and Gabapin-300) was spiked with pure GBP at three different concentration levels (50, 100, and 150% of the quantity present in the capsule powder), and the total was analyzed by the proposed methods. The results of this study are presented in Table 7 and indicate that the excipients present in the capsules did not interfere in the assay. 

## 4. Conclusions

Two simple, rapid, sensitive, and selective spectrophotometric methods have been proposed for the analysis of GBP in pure form and in capsules. The proposed methods utilized picric acid and 2,4-dinitrophenol as analytical reagents for the determination of GBP based on the formation of ion-pair complexes with the drug. From Sandell's sensitivity and LOD data presented in Table 3, it is clear that the method A (using PA) is more sensitive than method B (using 2,4-DNP). This is due to the fact that the proton of the hydroxyl group in PA is more reactive or more acidic, towards amino group of GBP, compared with the same in 2,4-DNP. The high reactivity of the replaced proton in PA than in 2,4-DNP can be attributed to the presence of more number of electron-withdrawing (nitro) groups in PA. The proposed methods are superior to all chromatographic methods [6–11], most visible spectrophotometric methods [16–19], and the automated spectrophotometric method [22] reported so far for analysis of GBP in terms of its sensitivity. Moreover, the proposed methods are free from the usual analytical complications like heating or extraction steps and use inexpensive and easily available chemicals and instrument. Hence, the proposed methods can be readily adopted by pharmaceutical quality control laboratory for routine analysis.

## Figures and Tables

**Table 1:** Chromatographic methods reported for the determination of GBP in pharmaceuticals.

Technique	Chromatographic conditions			LOD, mg mL^−1^	Range, *μ*g mL^−1^	Ref.
	Mobile phase	Flow rate, mL min^−1^	Detection, UV, nm			
(1) HPLC	Ammonium dihydrogen orthophosphate buffer and methanol in 60 : 40 (v/v)	1.0	200	NR	2500–7500	[6]
(2) HPLC	Methanol-acetonitrile- potassium dihydrogen phosphate (pH 5.2; 0.028 M) (25 : 10 : 65, v/v)	1.0	210	NR	100–3800	[7]
(3) HPLC	Acetonitrile-sodium dihydrogenphosphate (pH 2.5; 0.05 M) (70 : 30, v/v)	1.5	360	NR	10–500	[8]
(4) HPLC	Methanol-potassium dihydrogen orthophosphate solution (20 : 80, v/v) containing 10% NaOH	1.0	275	NR	940–1060	[9]
(5) HPLC	Acetonitrile-10 mM KH_2_PO_4_/10 mM K_2_HPO_4_ (pH 6.2) (8 : 92, v/v)	1.0	210	0.005	500–5000	[10]
(6) HPLC	Methanol-acetonitrile-20 mM KH_2_PO_4_ (pH 2.2) (5 : 5 : 90, v/v/v)	1.25	210	0.015	50–650	[11]

NR: Not reported.

**Table 2:** Comparison of the proposed and the existing visible spectrophotometric methods.

Sl. No.	Reagent/s used	Methodology	*λ*_ max_ (nm)	Linear Range, *μ*g mL^−1^ and *ε*, l mol^−1^ cm^−1^	LOD, *μ*g mL^−1^	Reaction time, min	Remarks	Ref.
(1)	(a) Vanillin	Condensation product measured	376	80–360 (*ε* = 4.57 × 10^2^)	NR	30	Less sensitive, measurements at shorter wavelengths for (a) and (c), heating required for (b) and (c).	
	(b) Ninhydrin	Condensation product measured	569	40–280 (*ε* = 5.16 × 10^2^)	NR	5		[16]
	(c) p-benzoquinone	Condensation product measured	369	80–320 (*ε* = 4.63 × 10^2^)	NR	5		
(2)	Acetylacetone and formaldehyde	Condensation product measured	415	20–140 (*ε* = 1.66 × 10^3^)	NR	20	Heating required, less sensitive	[17]
(3)	(a) Iodine	Triiodide ion measured	360	6–30 (*ε* = 6.19 × 10^3^)	0.39	—	Shorter wavelength and less sensitive (a) Expensive reagent used (b) Less sensitive	
	(b) 7,7,8,8- tetracyano- quinodimethane	Radical anions measured	842	8–24 (*ε* = 7.22 × 10^3^)	0.48	20		[18]
	(c) DDQ	-do-	456	12–36 (*ε* = 9.34 × 10^3^)	1.20	—		
	(d) Chloranilic Acid	-do-	535	60–200 (*ε* = 7.19 × 10^3^)	7.59	—		
	(e) Tetracyano ethylene	-do-	412	40–140 (*ε* = 1.10 × 10^3^)	3.54	15		
	(f) Chloranil	-do-	521	40–120 (*ε* = 1.23 × 10^3^)	3.33	20		
(4)	Ninhydrin	Colored product measured	405	50–300	NR	5	Heating required, less sensitive	[19]
(5)	(a) Ninhydrin	Condensation product measured	568	2–30 (*ε* = 1.25 × 10^4^)	0.15	20		[20]
	(b) 7,7,8,8- tetracyano- quinodimethane	Charge transfer complex measured	439	4–30 (*ε* = 6.77 × 10^4^)	0.04	15	Heating require Expensive reagent used.	
(6)	NQS	Automated flow injection using piezoelectric pumping	480	Up to 150	11.0 and 9.8	—	Less sensitive and complicated experimental setup	[22]
(7)	(a) Picric acid	Ion-pair complex measured	415	1.25–15 (*ε* = 1.09 × 10^4^)	0.23	10	Simple, sensitive selective, no heating step and inexpensive reagents used.	This work
	(b) 2,4-Dinitrophenol	-do-	420	2–18 (*ε* = 0.64 × 10^4^)	0.75	10		

DDQ: 2,3-dicloro-5,6-dicyano-1,4-benzoquinone, NQS: Sodium 1,2-naphthoquinone-4-sulfonate, NR: Not reported.

**Table 3:** Regression and analytical parameters.

Parameter	Method A	Method B
*λ*_ max_, nm	415	420
Beer's law limits (*μ*g mL^−1^)	1.25−15	2−18
Molar absorptivity (l mol^−1^ cm^−1^)	1.09 × 10^4^	0.64 × 10^4^
Sandell sensitivity* (*μ*g cm^−2^)	0.0158	0.0267
Limit of detection (*μ*g mL^−1^)	0.23	0.75
Limit of quantification (*μ*g mL^−1^)	0.71	2.28
Regression equation, Y**=	0.0072 + 0.062X	0.0094 + 0.035X
Correlation coefficient (r)	0.9993	0.9994
Standard deviation of intercept (S_a_)	0.0229	0.0276
Standard deviation of slope (S_b_)	0.0025	0.0026

*Limit of determination as the weight in *μ*g per mL of solution, which corresponds to an absorbance of A = 0.001 measured in a cuvette of cross-sectional area 1 cm^2^ and l = 1 cm. **Y = *a* + *bX*, where Y is the absorbance, a is the intercept, b is the slope, and X is the concentration in *μ*g mL^−1^.

**Table 4:** Precision and accuracy.

Method	GBP taken (*μ*g mL^−1^)	Intraday (*n* = 7)			Interday (*n* = 5)		
		GBP found^a^ (*μ*g mL^−1^)	%RSD^b^	%RE^c^	GBP found^a^ (*μ*g mL^−1^)	%RSD^b^	%RE^c^
Method A	5.00 7.50 10.00	5.13 7.66 10.28	1.54 1.03 1.72	2.60 2.13 2.80	5.16 7.72 10.36	1.42 1.65 2.11	3.20 2.93 3.60

Method B	8.00 12.00 16.00	8.18 12.23 15.82	1.46 2.34 1.98	2.25 1.92 −1.12	8.23 12.28 15.76	1.77 2.59 2.45	2.88 2.33 −1.50

^a^Mean value of *n* determinations.

^b^Relative standard deviation (%).

^c^Bias (%): [(found − taken)/taken] × 100.

**Table 5:** Method robustness and ruggedness.

Method	GBP Taken *μ*g mL^−1^	Robustness (%RSD)		Ruggedness (%RSD)	
		Reagent volume	Reaction time	Interanalysts (*n* = 3)	Interinstruments (*n* = 3)
	5.00	1.48	0.76	0.58	2.58
Method A	7.50	1.26	0.85	0.63	1.74
	10.00	0.85	1.08	0.87	2.64

	8.00	0.68	1.18	0.75	3.03
Method B	12.00	1.12	1.26	1.09	2.86
	16.00	1.38	1.56	1.27	3.25

*In method A, the volume of PA was 0.8, 1.0 and 1.2 mL, and the reaction time was 8, 10, and 12 min. In method B, the volume of 2,4-DNP added was 0.65, 0.75, and 0.85 mL, and the reaction time was 8, 10, and 12 min.

**Table 6:** Results of assay of capsules and statistical evaluation.

Capsule brand name	Found (% of nominal amount ± SD)*		
	Reference method	Proposed methods	
		Method A	Method B
Gabantin-100	99.32 ± 1.04	98.27 ± 1.97 *t* = 1.05 *F* = 3.59	97.01 ± 2.24 *t* = 2.09 *F* = 4.64

Gabapin-300	98.07 ± 1.37	99.13 ± 1.88 *t* = 1.02 *F* = 1.88	98.96 ± 2.11 *t* = 0.79 *F* = 2.37

*Mean value of five determinations.

Tabulated *t*-value at the 95% confidence level is 2.78.

Tabulated *F*-value at the 95% confidence level is 6.39.

**Table 7:** Results of recovery study by standard addition method.

Capsules studied	Method A				Method B			
	GBP in capsule, *μ*g mL^−1^	Pure GBP added, *μ*g mL^−1^	Total found, *μ*g mL^−1^	Pure GBP recovered*, Percent ± SD	GBP in capsule, *μ*g mL^−1^	Pure GBP added, *μ*g mL^−1^	Total found, *μ*g mL^−1^	Pure GBP recovered*, Percent ± SD
Gabantin-100	4.91 4.91 4.91	2.50 5.00 7.50	7.36 9.95 12.36	98.00 ± 2.82 100.8 ± 1.96 99.33 ± 2.44	3.88 3.88 3.88	2.00 4.00 6.00	5.91 7.84 9.81	101.5 ± 1.74 99.01 ± 2.09 98.83 ± 2.62

Gabapin-300	5.95 5.95 5.95	3.00 6.00 9.00	9.00 11.84 15.17	101.7 ± 2.36 98.16 ± 2.28 102.4 ± 2.73	3.95 3.95 3.95	2.00 4.00 6.00	5.96 7.87 10.06	100.5 ± 2.41 98.02 ± 2.09 101.8 ± 2.18
